# 157. Impact of Pharmacist-Generated Oral Antimicrobial Test Prescription on Discharge Medication Access and Outcome

**DOI:** 10.1093/ofid/ofab466.157

**Published:** 2021-12-04

**Authors:** Surafel Mulugeta, Susan L Davis, Susan L Davis, Rachel Kenney

**Affiliations:** 1 Henry Ford Hospital, Detroit, MI; 2 Wayne State University, Detroit, MI

## Abstract

**Background:**

Cost barriers to accessing discharge oral antimicrobials (ABX) may delay discharges and result in suboptimal discharge ABX. Use of electronic test prescriptions (eTP) or “price checks” is controversial due to potential for erroneous dispensing. This study evaluated discharge ABX access and outcome after implementation of a standardized, inpatient pharmacist-initiated ABX eTP process in collaboration with discharge pharmacy.

**Methods:**

IRB approved, retrospective, cross-sectional cohort pilot-study. Inclusion: home bound adults admitted for ≥ 72 hours from 1/1/18-2/28/19 and discharged on oral ABX. Patients with an ABX eTP prior to discharge were compared to those discharged on ABX but no eTP. Data were reported using descriptive statistics and bivariate analysis. Primary endpoint: discharge delay after medical stability. Secondary endpoints: medication access, unplanned encounters, and % of patients discharged on first-line ABX.

**Results:**

84 patients included: 43 no-ETP and 41 eTP. 75 ABX eTP evaluated among 41 patients. Patients in the no-eTP group had higher Charlson comorbidity index (*P =* 0.004) and immunosuppression (24% vs. 12%; *P =* 0.014). Median length of stay, days: 6 (5 – 9) eTP vs. 8 (5 – 15) no-eTP (*P =* 0.026). Most common eTP requested by pharmacist: linezolid (17, 23%) and oral vancomycin (12, 16%) (Figure 1). eTP results were documented in the medical record in < 24 hours for 66 (88%) of inquiries. 49 (65%) prescriptions were approved by insurance; 16 (21%) had no out of pocket cost and 8 (11%) required prior authorization (PA) (Table 1). Linezolid (5, 35%) and public insurance (10, 71%) were frequently associated with barriers. 29 (70%) patients were discharged on the same ABX as the eTP. There were no discharge delays or erroneous dispensing. 14 (33%) no-eTP and 15 (37%) eTP patients experienced unplanned healthcare encounters after discharge. 9/84 (11%) patients were discharged on suboptimal ABX. Non-white race 8/9 (89%) *P* = 0.047 and public insurance 8/9 (89%) *P* = 0.063 were associated with suboptimal discharge ABX.

Figure 1. Oral Antimicrobial Test Prescription Pattern (n=75)

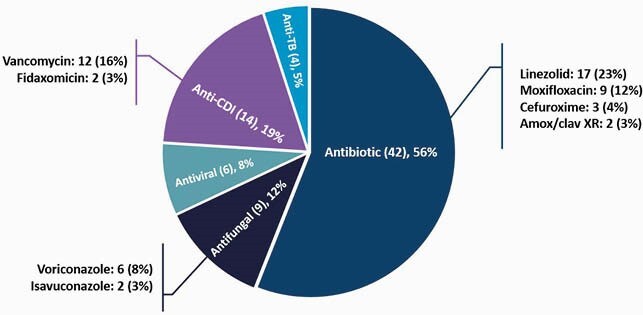

Table 1. Oral Antimicrobial Test Prescription Result (n=75)

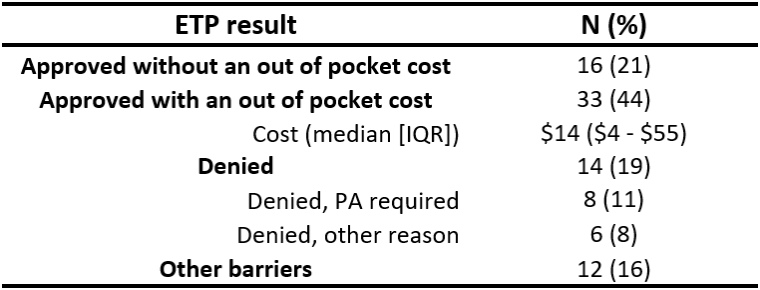

**Conclusion:**

A standardized eTP process appears to be a safe way to evaluate out of pocket cost without prolonging length of stay. Future work will focus on inequity in access to first line ABX.

**Disclosures:**

**Susan L. Davis, PharmD**, Nothing to disclose **Rachel Kenney, PharmD**, **Medtronic, Inc.** (Other Financial or Material Support, spouse is an employee and shareholder)

